# The case for philanthropic investment to increase colorectal cancer screening rates: A novel paradigm to address a public health challenge

**DOI:** 10.1002/cam4.2745

**Published:** 2019-12-05

**Authors:** Ariel Carmeli, Lee Dranikoff, Arnab Kundu, Uri Ladabaum

**Affiliations:** ^1^ Biden Cancer Initiative Washington DC USA; ^2^ Colorectal Cancer Alliance Board of Directors Washington DC USA; ^3^ American Securities New York NY USA; ^4^ Clarify Health Solutions New York NY USA; ^5^ Division of Gastroenterology and Hepatology Stanford University School of Medicine Stanford CA USA

**Keywords:** health economics, philanthropy, screening participation

## Abstract

**Background:**

Colorectal cancer (CRC) remains a leading cause of cancer‐related death despite being highly preventable. Efforts to increase participation in CRC screening have not met national goals. We developed a novel approach: building a business case for philanthropic investment in CRC screening.

**Methods:**

A taskforce representing the public health community, professional societies, charitable foundations, academia, and industry was assembled to: (a) quantify the impact of improving CRC screening rates; (b) identify barriers to screening; (c) estimate the “activation cost” to overcome barriers and screen one additional person; (d) develop a holistic business case that is attractive to philanthropists; and (e) launch a demonstration project.

**Results:**

We estimated that of 50 600 CRC deaths annually in the US, 55% occur in 50‐ to 85‐year‐olds and are potentially addressable by improvements in CRC screening. Barriers to screening were identified in all patient journey phases, including lack of awareness or insurance and logistical challenges in the pre‐physician phase. The cost to activate one person to undergo screening was $25‐175. This translated into a cost of $6000‐36 000 per CRC death averted by philanthropic investment. Based on this work, the Colorectal Cancer Alliance launched the effort “March Forth” to prevent 100 000 CRC deaths in the US over 10 years, with the first pilot in Philadelphia.

**Conclusions:**

A holistic business plan can attract philanthropy to promote CRC screening. A simple message of “You can save a life from CRC with a $25 000 donation” can motivate demonstration projects in regions with high CRC rates and low screening participation.

## Graphical abstract

Although colorectal cancer (CRC) is highly preventable with screening, efforts to increase screening participation have not met national goals. We present the work of a taskforce of multiple stakeholders to devise a novel strategy: developing a holistic business case for philanthropic investment in CRC screening, focused on paying for the cost to motivate an unscreened person to get screened. Based on this work, the Colorectal Cancer Alliance, the largest advocacy organization for CRC in the US, launched the effort “March Forth” to prevent 100 000 CRC deaths in the US over 10 years, with the first pilot in Philadelphia.

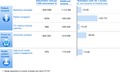

## 1 INTRODUCTION

Screening decreases colorectal cancer (CRC) incidence and mortality^1^ and is highly cost‐effective.^2^ Despite the effectiveness of CRC screening and the availability of a range of screening modalities, including colonoscopy and fecal immunochemical testing (FIT), approximately one‐third of the screen‐eligible US population does not undergo regular CRC screening^3^ and CRC remains the second leading cause of cancer death in the US, resulting in approximately 50 000 deaths per year.^4^

Multiple barriers to CRC screening have been identified, and while some US health systems have shown success in addressing these barriers,^5^, ^6^ these efforts have largely not been replicated across the country. Recognizing a plateau in CRC screening rates by 2013, Dr Howard Koh issued the challenge to develop a bold goal for CRC screening, resulting in the launch of the “80 by 18” campaign, which aimed to increase CRC screening in the US from approximately 65% in 2014 to 80% by the end of 2018.^7^

In 2016, one of the authors (LD) recognized the potential opportunity in developing a business case for philanthropic investment, focused on paying for the cost to motivate an unscreened person to get screened, as a novel approach to increase CRC screening in the US. In support of the American Cancer Society (ACS) and National Colorectal Cancer Roundtable (NCCRT) “80 by 18” campaign and to generate a “call to action” for private and public foundations, a taskforce of multiple stakeholders was convened in 2016 in a pro bono initiative sponsored by the Colorectal Cancer Alliance (“Alliance”). The group included members from the Alliance, ACS, NCCRT, Entertainment Industry Foundation (EIF), public health community, academia, and volunteers from McKinsey & Company.

The aims of the call to action are to substantially increase philanthropic investment in CRC screening and prevention, and to apply these funds to high‐yield efforts that will rapidly narrow the gap between the current nationwide CRC screening rate and the target goal of 80%.

This paper presents the results of the taskforce's work in five domains:

1. Quantifying the potential public health impact of improving CRC screening rates.
2. Identifying barriers to screening and their relative contribution to screening nonadherence.
3. Estimating the “activation cost” to overcome barriers and screen one additional person.
4. Developing a holistic business case that is attractive to philanthropic organizations, based on the estimated cost to prevent a death from CRC.
5. Launching a demonstration project in the city of Philadelphia that embodies this novel approach.

This development of a business‐mindset approach to CRC screening, including estimation of return on investment, may help local and regional efforts to improve CRC screening in the US and serve as a roadmap for efforts to address other major public health problems.

## 2 METHODS

### 2.1 Overall approach and support for this effort

The initial work to build the business case took place over a 2‐month period in June‐August 2016. Research, analysis, and discussion were coordinated weekly, and two working sessions were convened in Washington DC with representation from ACS, the Alliance, EIF, academia, medical and public health experts in CRC screening and prevention, and volunteer staff of McKinsey & Company (see Acknowledgments).

### 2.2 Quantifying the potential public health impact of improving CRC screening rates

The number of CRC deaths that could be prevented by improved CRC screening rates was estimated based on epidemiological data and previous research studies.

The US Preventive Services Task Force recommends routine CRC screening between the ages of 50 and 75.^1^ The ACS recently made the qualified recommendation to begin screening at age 45.^8^ This work predated that recommendation, which explains our focus on persons 50 and older. Because the clinical effectiveness of programs based on colonoscopy may be comparable to those based on FIT,^2^ our estimates relied on assumptions about colonoscopy for simplicity.

The total number of CRC deaths in the US annually was based on Surveillance, Epidemiology, and End Results (SEER) program data. We categorized this annual total into three mutually exclusive groups: (A) Deaths among people aged <50 or >84 (outside typical screening ages); (B) Deaths among people aged 50‐84 that are not “addressable” by increased screening; and (C) Deaths among people aged 50‐84 that are “potentially addressable” by increased screening. We defined a “potentially addressable” CRC death as one occurring in a person who was not adherent with screening and in whom the CRC death could have potentially been prevented by screening. Group A was estimated directly from SEER. Group B was estimated as all deaths from interval cancers and a portion of the deaths from screen‐detected cancers. We assumed that routine screening occurs at ages 50‐75, and we made the conservative assumption that deaths might be averted through age 85.

We estimated the total number of deaths from interval cancers as follows:

$$\begin{array}{cc} & \text{US}\;\text{population}\;\text{aged}\;50-84\;\text{compliant}\;\text{with}\;\text{screening}\\ & \;\times \text{Rate}\;\text{of}\;\text{interval}\;\text{cancer}\times \text{Deaths}\;\text{per}\;\text{interval}\;\text{cancer}\\ & \;=\text{Total}\;\text{number}\;\text{of}\;\text{interval}\;\text{cancer}\;\text{deaths}. \end{array}$$

We estimated the US population fraction aged 50‐84 that is adherent with screening by multiplying the population screening compliance rate^3^ by the US population aged 50‐84.^9^ We estimated the rate of interval cancer as 5 cases per 10 000 person‐years based on data from Corley et al.^10^

We estimated the total number of deaths from screen‐detected CRCs as follows:

$$\begin{array}{cc} & \text{Number}\;\text{of}\;\text{people}\;\text{screened}\;\text{per}\;\text{year}\times \text{Rate}\;\text{of}\;\text{CRC}\\ & \;\times \text{Death}\;\text{rate}\;\text{for}\;\text{screen-detected}\;\text{cancers}\\ & \;=\text{Total}\;\text{number}\;\text{of}\;\text{screen-detected}\;\text{cancer}\;\text{deaths}. \end{array}$$

We calculated the above separately for first‐time screens and for later screens. We assumed that in any year, one‐quarter of people screened with colonoscopy are first‐time screeners, based on an estimated 3‐4 colonoscopies per person over a lifetime. We assumed that the number of people screened with colonoscopy per year was one‐tenth of the US population aged 50‐84 adherent with screening, based on an estimate of one colonoscopy every 10 years, reflective of screening guidelines.

We based the rate of finding CRC during a first‐time screen on colonoscopy screening data from Imperiale et al.^11^ That study reported 65 cases of CRC among 9989 people screened. We assumed that the likelihood of finding CRC in subsequent screens is lower, and estimated this relative reduction using data from Singh et al.^12^ We estimated the death rate for screen‐detected CRCs based on stage distribution,^11^ and stage‐specific mortality.^13^

Screen‐detected deaths can occur among people who were not previously adherent, for example, waited until age 55 for the first CRC screen, and it is possible that some of these deaths might have been prevented by timely screening. A 12‐month delay in colonoscopy after an abnormal FIT results in a 3.2 odds ratio for advanced disease,^14^ and we thus estimated that approximately one‐third of screen‐detected CRC deaths are not addressable by a CRC screening initiative. We conducted sensitivity analyses assuming that 75% or 100% of these screen‐detected CRC deaths are not addressable.

Group C was estimated by subtracting Groups A and B from the total annual CRC deaths in the US

Details of the calculations are provided in the Appendix.

### 2.3 Identifying barriers to screening and their relative contribution to screening nonadherence

We identified the most significant barriers to adherence to CRC screening guidelines, and quantified the approximate magnitude for each barrier, based on a review of published literature and publicly available data from the ACS and the Centers for Disease Control and Prevention (CDC) as shown in Table A1. The primary barriers identified were as follows: (1) lack of insurance; (2) lack of patient awareness of CRC screening guidelines; (3) logistical challenges for patient to get screened; (4) lack of primary care provider (PCP) recommendation for screening; (5) patient avoidance despite PCP recommendation; (6) challenges with colonoscopy preparation, and (7) lack of timely follow‐up after initial screening. These barriers were further grouped into phases along a patient's journey: Pre‐physician (1‐3), screening recommendation (4), post‐recommendation through completion of screening (5‐6), and follow‐up (7).

We recognize that multiple barriers may overlap, but in order to inform future screening campaigns, we aimed to derive simplified estimates for the principal population impact of each barrier. The overall contribution of each barrier was first expressed as the percent of the not up‐to‐date population affected by a given barrier, based on the mid‐point of the ranges reported (Table A1). We then normalized the total impact of all the barriers to sum up to the current fraction of the population that is not up‐to‐date with CRC screening.

### 2.4 Estimating the “activation cost” to overcome barriers and screen one additional person

**Figure 1:**
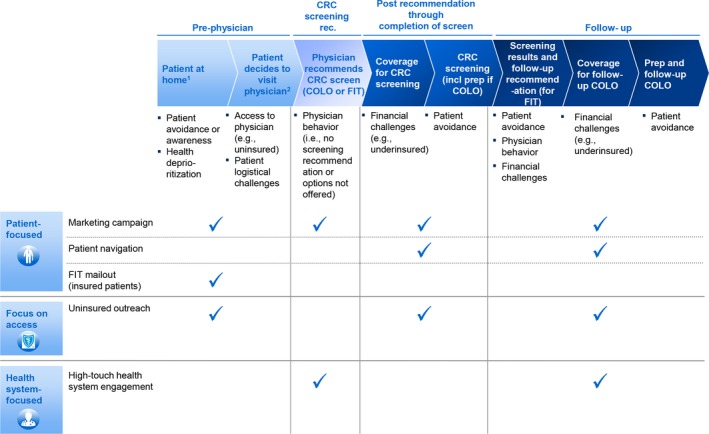
Patient journey, barriers, and potential impact of interventions. CRC, colorectal cancer; FIT, fecal immunochemical testing

We held discussions in the two working sessions to prioritize the set of screening interventions that a CRC screening campaign should focus on. Five interventions were prioritized because they together target different barriers (Figure 1) and have individually been proven to increase CRC screening. For each intervention, published literature and expert opinions were used to estimate the potential impact and cost (Table A2). Interviews were conducted with experts familiar with each intervention: from NYC C5 (Citywide Colon Cancer Control Coalition) for patient navigation, Kaiser Permanente for FIT mailout, UC San Diego for uninsured outreach, and ACS for high‐touch health system engagement. The five interventions were:

1. *Marketing campaign*: National and micro‐targeted media campaigns that emphasize “no best test” and provide information on where and how to get screened. Based on previous CDC smoking cessation marketing campaigns,^15^ we estimated that 8%‐10% of the unscreened population might undergo CRC screening as a result of the campaign.
2. *Patient navigation*: A patient navigator provides personal guidance to patients as they move through completion of colonoscopy. Navigation has led to ~20%‐29% increases in colonoscopy completion rates in health systems.^16^, ^17^
3. *FIT mailout*: Partnership with a payor or health system to mail FIT kits to the homes of unscreened, insured individuals. This intervention has led to ~20%‐48% FIT completion rate among previously nonadherent people.^18^
4. *Uninsured outreach*: Intervention, such as FIT mailout, is targeted to uninsured individuals, and the cost of FIT and subsequent colonoscopies are covered. This intervention has led to ~40%‐60% FIT completion rate among previously noncompliant people.^19^, ^20^
5. *High‐touch health system engagement*: Dedicated field staff engages health systems around the country to share CRC screening best practices. Given that PCPs have very limited time with each patient, the focus is to help health systems find ways to educate patients outside of a PCP appointment (eg, educational tools; nurse consult). Such interventions have led to ~4%‐15% increases in CRC screening rates in select populations (ACS, personal communication)

For each intervention, we used the above estimates of impact to calculate the “activation cost” per person, defined as the cost to motivate a person not currently participating in CRC screening to participate. Our calculations of activation cost do not include medical or treatment costs, with the exception of the uninsured outreach intervention, which includes the cost for a follow‐up colonoscopy when needed. Advocacy or lobbying were not considered. For the marketing campaign, the target population cohort was estimated at national scale, and for the other four interventions, we considered groups of 1000 unscreened people. The details of the “activation cost” calculations were as follows:

1. Marketing campaign: TV airtime cost for a large public health campaign, based on CDC's national “Smoking Kills” campaign, *divided by* the number of people motivated to undergo screening.
2. Patient navigation: Sum of the costs to navigate patients with referral to colonoscopy, plus the costs to manage the navigation program, *divided by* the incremental colonoscopy completion rate due to patient navigation.
3. FIT mailout: Sum of the costs to mail FIT kits, plus the costs to navigate people who test positive through colonoscopy, *divided by* the number of people who return the FIT kit.
4. Uninsured outreach: Sum of the costs to identify unscreened people who are also uninsured, plus the costs of FIT kits and mailing, plus the costs of the follow‐up colonoscopy for people who test positive, *divided by* the number of people who return the FIT kit.
5. High‐touch health system engagement: Sum of salaries for a field force required to engage with the set of PCPs who collectively care for 1000 unscreened patients *divided by* the number of unscreened patients who become screened as a result of the engagement.

There may be additional cost components not included in the calculations described above. However, our working session attendees believe that the included costs represent the majority of costs and our estimates are reasonable reflections of total activation cost.

### 2.5 Developing a holistic business case that is attractive to philanthropic organizations

We estimated the cost per CRC death prevented with the aim of garnering interest from philanthropic organizations. This was based on the estimated activation costs, and the reductions in CRC deaths with screening. We took a conservative approach and used the high end of the estimated range for each activation cost.

The frequency of testing required to remain compliant with screening varies by screening modality. We focused on the two most commonly used screening modalities, colonoscopy and FIT. Over 10 years, FIT activation needs to occur 10 times, while one‐time activation suffices for colonoscopy. Based on experience of health care professionals who have managed FIT mailout programs, we assumed that health systems or payors will have incentives to continue FIT programs once they are launched and their benefits are appreciated. Thus, we assumed that a philanthropic investor would need to cover only 30% of the repeat activation cost for FIT in years 2‐10.

### 2.6 Launching a demonstration project in the city of Philadelphia

We selected Philadelphia as the location for a demonstration project for CRC screening because it has a relatively large population among major cities in the US and access to world‐class health care institutions. Since the completion of the research and analysis phase, foundational work with the Alliance and local organizations in Philadelphia has ensured a successful transition to a pilot program phase, during which the implementation plan for a CRC screening initiative in Philadelphia has been developed.

## 3 RESULTS

### 3.1 Quantifying the potential public health impact of improving CRC screening rates

**Figure 2:**
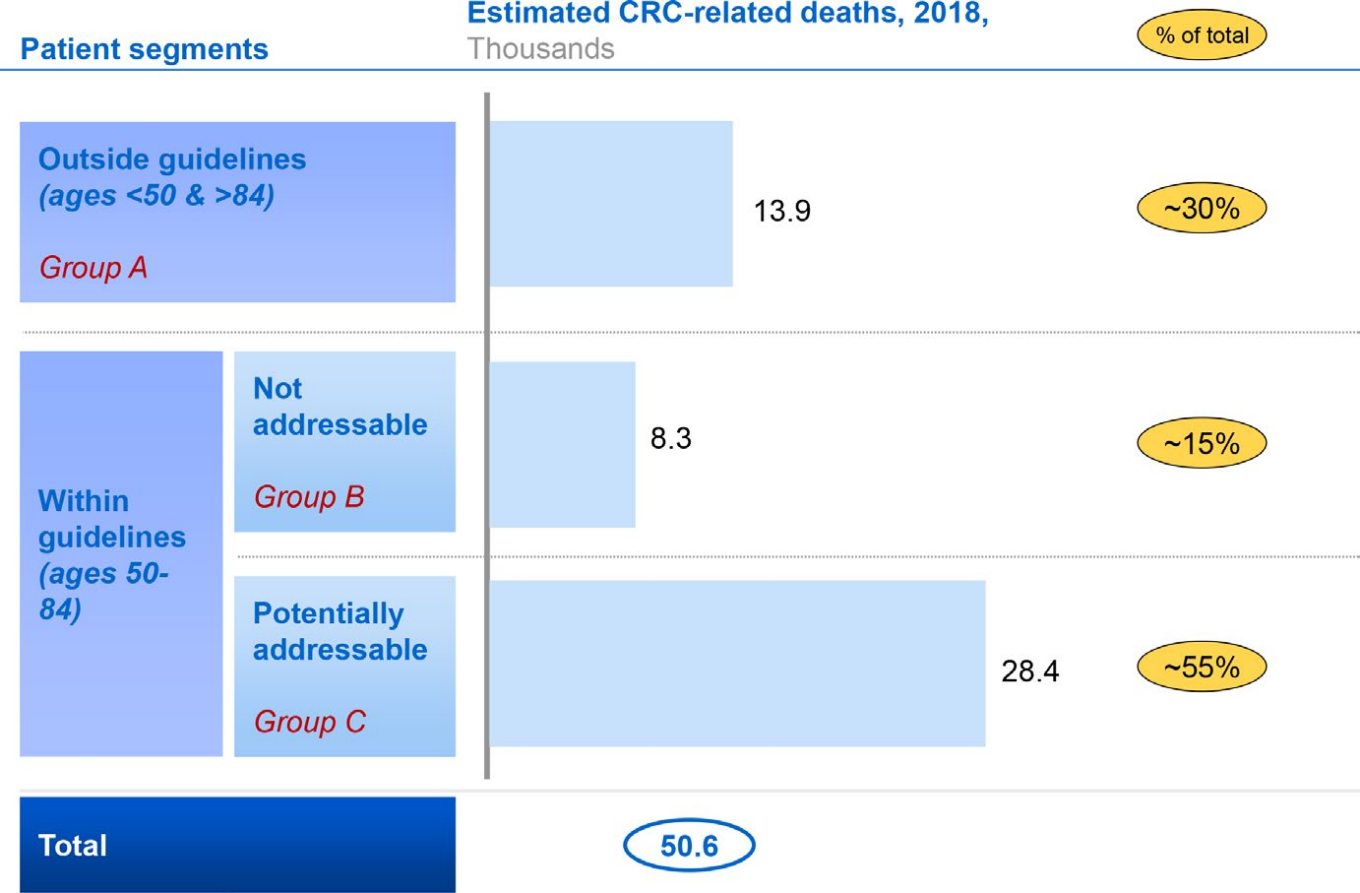
Annual estimated colorectal cancer deaths, by age and screening status. CRC, colorectal cancer

Of the approximate 50 600 CRC deaths in 2016 in the US, approximately 28 400 (55%) were estimated to be potentially addressable by a CRC screening initiative in persons aged 50‐84 (group C) (Figure 2; Appendix). If we assumed that 75% of screen‐detected CRC deaths are not addressable, the estimate decreased to 26 500 (52%). If we assumed that 100% of screen‐detected CRC deaths are not addressable, the estimate decreased to 25 400 (50%).

### 3.2 Identifying barriers to screening and their relative contribution to screening nonadherence

**Figure 3:**
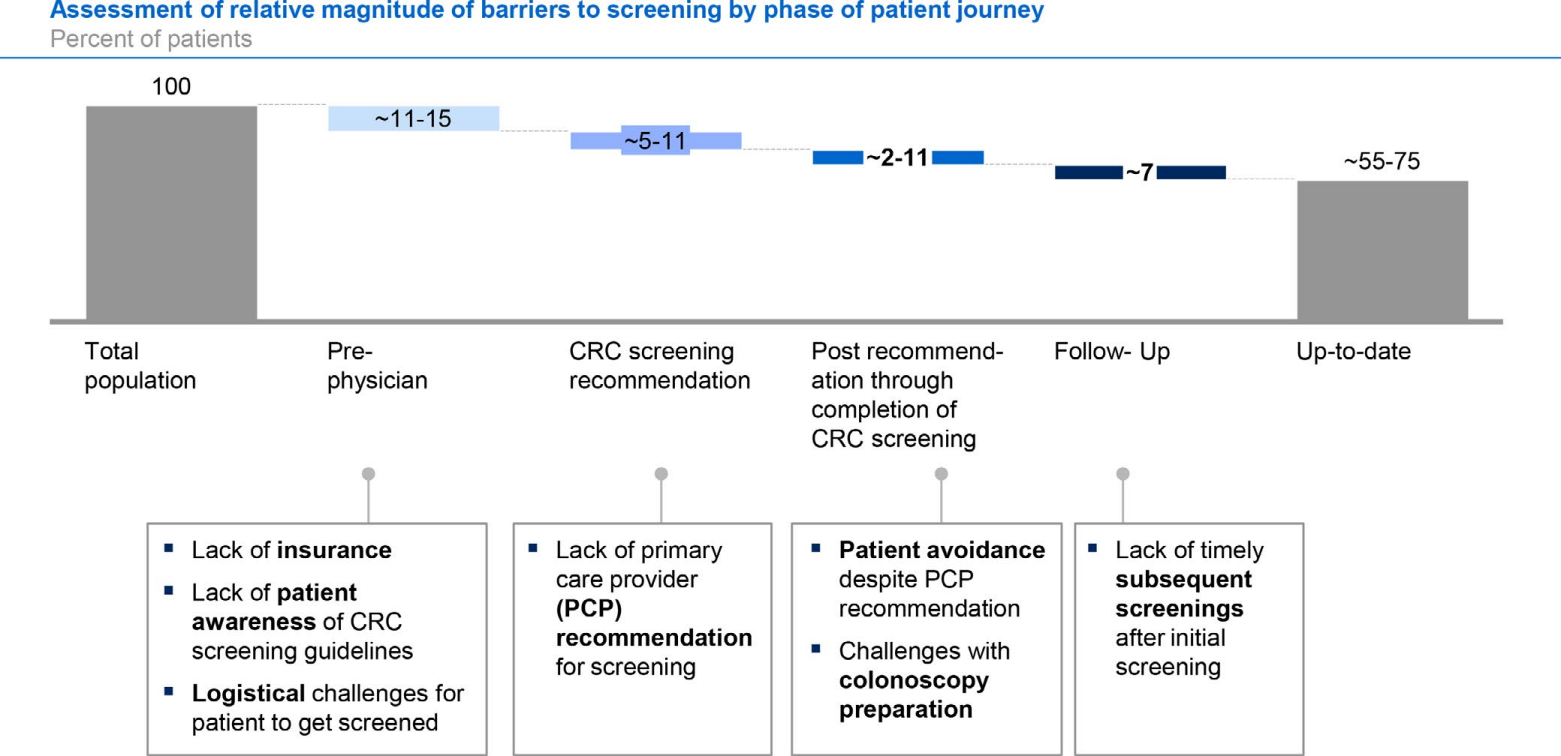
Barriers to screening by phase. CRC, colorectal cancer

Figure 3 illustrates the estimated relative magnitude of barriers in the four phases of a person's screening journey. The largest barriers to CRC screening were estimated to be in the pre‐physician phase, including lack of insurance, awareness, and logistical challenges. These affected 11%‐15% of the population. The fraction of the population affected by barriers in other phases was estimated as 5%‐11% in the screening recommendation phase, 4%‐13% in the post‐recommendation through completion phase, and 6% in the follow‐up phase.

### 3.3 Estimating the “activation cost” to overcome barriers and screen one additional person

**Figure 4:**
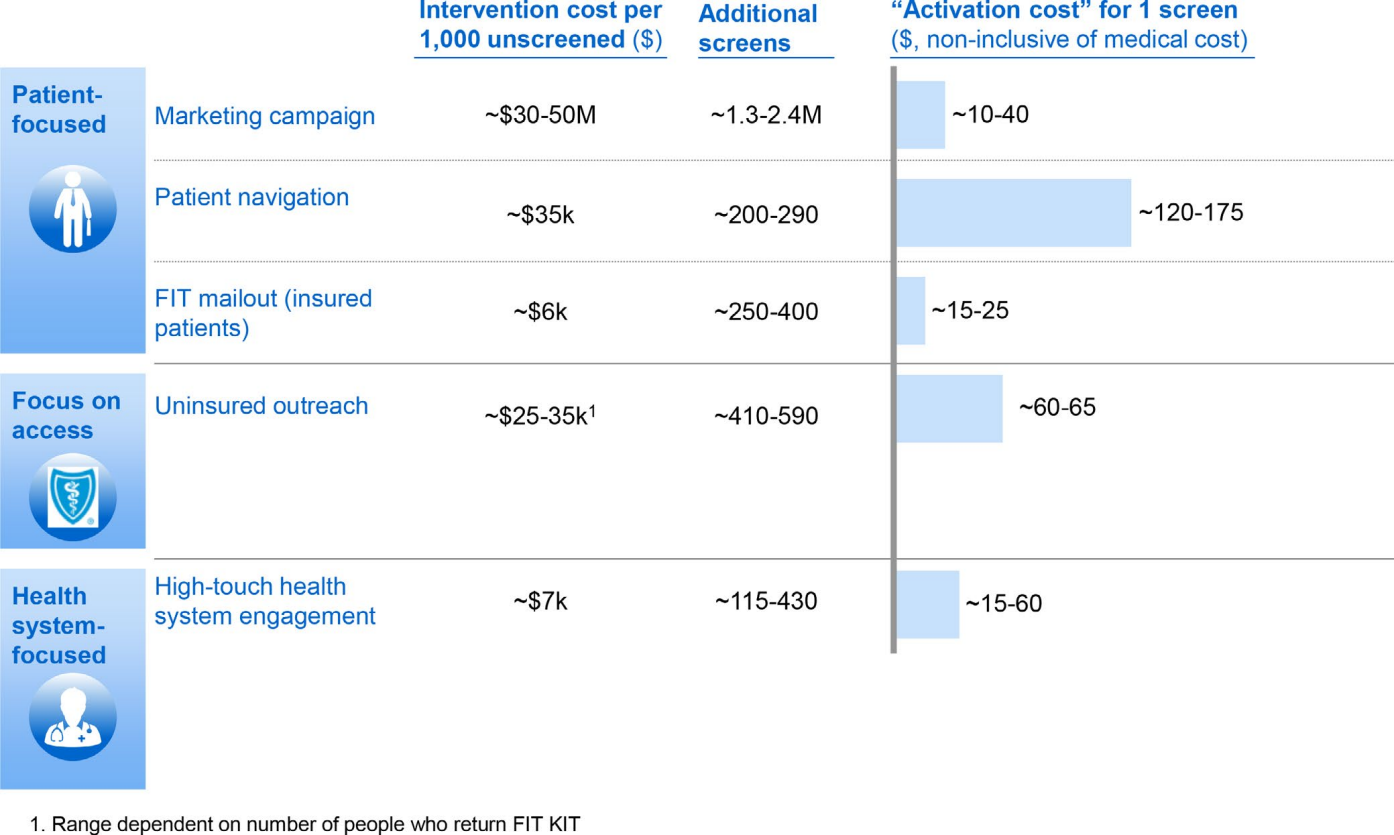
Activation costs of the five prioritized interventions. FIT, fecal immunochemical testing

Figure 4 shows the estimated activation cost with each of the five prioritized interventions. FIT mailout was estimated to have the lowest activation cost (range of $15‐25 per additional person screened) and patient navigation for colonoscopy the highest (range of $120‐175 per additional person screened).

### 3.4 Developing a holistic business case that is attractive to philanthropic organizations

We estimated a potential 84% reduction in CRC mortality in currently unscreened persons (group C) who take up screening (details in Appendix). Assuming this 84% reduction in CRC mortality, we estimated that approximately 6.8 CRC deaths could be prevented over 10 years for every 1000 people who take up screening (Appendix). On a national scale, this translates to approximately 100 000 CRC deaths prevented over 10 years if half of today's unscreened population, ~15 million people, were activated to be screened.

The estimated costs per CRC death prevented for each intervention, and their derivation, are shown in Table 1. The costs ranged from approximately $6000 to $36 000. Under the sensitivity analysis that assumes no screen‐detected CRC cancer deaths are addressable, the costs ranged from $7000 to $44 000.

**Table 1:** Cost per death prevented by intervention

Marketing campaign					FIT mailout		Patient navigation	
Colonoscopy		FIT			FIT only		Colonoscopy only	
Activation cost (1 person)	$40	Activation cost (1 person)		$40	Activation cost (1 person)	$25	Activation cost (1 person)	$175
Activation cost (1000 people)	$40 000	Activation cost (1000 people)		$40 000	Activation cost (1000 people)	$25 000	Activation cost (1000 people)	$175 000
		Year 1 cost		$40 000	Year 1 cost	$25 000		
		Years 2‐10 cost		$108 000	Years 2‐10 cost	$67 500		
		Yearly cost		$40 000	Yearly cost	$25 000		
		% we cover (years 2‐10)		30%	% we cover (years 2‐10)	30%		
		Number of years		9	Number of years	9		
Total cost in 10 y	$40 000	Total cost in 10 y		$148 000	Total cost in 10 y	$92 500	Total cost in 10 y	$175 000
Deaths prevented over 10 y	6.8	Deaths prevented over 10 y		6.8	Deaths prevented over 10 y	6.8	Deaths prevented over 10 y	6.8
Cost per death prevented	$5924	Cost per death prevented		$21 918	Cost per death prevented	$13 699	Cost per death prevented	$25 917

High‐touch health system engagement					Uninsured Outreach	
Colonoscopy		FIT			FIT only	
Activation cost (1 person)	$60	Activation cost (1 person)		$60	Activation cost (1 person)	$65
Activation cost (1000 people)	$60 000	Activation cost (1000 people)		$60 000	Activation cost (1000 people)	$65 000
					
		Year 1 cost		$60 000	Year 1 cost	$65 000
		Years 2‐10 cost		$162 000	Years 2‐10 cost	$175 500
		Yearly cost		$60 000	Yearly cost	$65 000
		% we cover (years 2‐10)		30%	% we cover (years 2‐10)	30%
		Number of years		9	Number of years	9
Total cost in 10 y	$60 000	Total cost in 10 y		$222 000	Total cost in 10 y	$240 500
Deaths prevented over 10 y	6.8	Deaths prevented over 10 y		6.8	Deaths prevented over 10 y	6.8
Cost per death prevented	$8886	Cost per death prevented		$32 878	Cost per death prevented	$35 617

Abbreviation: FIT, fecal immunochemical testing.

### 3.5 Launching a demonstration project in the city of Philadelphia

Starting in 2019, the Alliance has developed a foundational structure for a separately branded effort (“March Forth”) by establishing relationships with the major health systems, public health organizations, and business community in Philadelphia. A health professional advisory group has been formed and several initiatives are in the process of being launched, including an educational campaign for federally qualified health centers, a patient navigation program in conjunction with two health systems, and an employer educational program. The Alliance has also embarked on a major fundraising campaign with corporations, foundations, and individuals. Four primary areas of focus and priority for the Philadelphia pilot have included work in the following:

- *Governance Structure/organization*: The Alliance, the largest advocacy organization for CRC in the US, convened its Board of Directors and approved a major investment and strategic shift to making prevention one of its top focus areas. The Alliance hired a full‐time senior leader to manage this effort and established March Forth with a separate board comprised of senior health care and business executives. The Alliance is establishing a voluntary leadership structure which includes an implementation committee and a local leadership committee. The implementation committee will comprise multidisciplinary stakeholders including representatives from the various health systems, payers, community organization representatives, other not‐for‐profit entities, and representatives from the Department of Health. Several subcommittees will be formed to support development of an evaluation plan, health professional education, and other implementation efforts.
- *Fundraising/fundraising strategy*: A nationally recognized fundraising firm has been engaged to build a fundraising apparatus. This has resulted in initial funds of $2 million including a significant investment from Independence Blue Cross, a major Philadelphia payor, and from others with stakeholder interest in supporting the Philadelphia initiative. The continued fundraising strategy includes outreach to individuals, corporations, family foundations, and other potential stakeholders.
- *Launch in Philadelphia as “First city”*: On 15 May 2018, March Forth was publicly announced to the news media, Philadelphia leadership, industry partners, and thought leaders. A landscape analysis of CRC screening efforts in Philadelphia is being developed.
- *Developing a national patient navigation capability*: A key recommendation that emerged from the project's initial phase was to focus on patient navigation as a critical component to success. The March Forth leadership is in discussions with health systems nationwide to pilot a new navigation capability. This would include a central center to support navigation across the US.

## 4 DISCUSSION

CRC remains the cause of nearly 50 000 deaths per year in the US, many of which are preventable. We believe that the business case for philanthropy in CRC prevention is now clear: over 100 000 CRC deaths could potentially be prevented over 10 years in the US for a relatively low activation cost of approximately $25 000 per CRC death prevented. Since prevention of CRC death is estimated to gain multiple life‐years,^21^ this translates into a highly attractive cost per life‐year gained, given the commonly accepted willingness‐to‐pay threshold of $100 000 per life‐year gained in the US^22^

This project's goal was to develop an easily understood model focusing on patient activation costs and the ultimate yield in terms of CRC deaths averted. Sophisticated decision‐analytic models of CRC screening exist that include complexities regarding natural history, test performance characteristics, CRC treatment, and complication rates. However, such models risk appearing as black boxes to potential philanthropists. Nonetheless, comparison to the results of previous modeling can serve as external validation of our estimates in this project. In a previous cost‐effectiveness analysis of one‐time navigation for colonoscopy, navigation increased screening by an absolute 25% and resulted in 26 CRC deaths averted in a cohort of 10 000, at an incremental navigation cost of $158 000.^23^ From this, a cost per death averted of $6080 can be calculated. Similarly, in a previous cost‐effectiveness analysis of fecal‐based screening, patient support programs increased consistent FIT screening by an absolute 35% and resulted in 1687 CRC deaths averted in a cohort of 100 000, at an incremental patient support cost of $8 415 000.^2^ From this, a cost per death averted of $4990 can be calculated. These estimates are in the lower end of the range of estimates in the current project.

Increasing CRC screening is a challenge rooted in multiple barriers across the patient journey; thus, a multipronged and coordinated interventional approach is required to effect meaningful change. In several countries, these challenges are being addressed by national or regional health services. While such systems do not exist for most of the US population, individual health systems and programs in specific geographic locations have developed successful public health initiatives promoting CRC screening. In Kaiser Permanente Northern California, CRC screening rates increased from 37% to 82% in the commercial population and from 41% to 91% in the Medicare population from 2005 to 2015.^5^ This was driven primarily through two key levers: organizational commitment and a patient‐centered approach with a focus on FIT outreach. New York City's C5 was organized in 2003 by the New York City Department of Health and Mental Hygiene with the goals of increasing use of screening colonoscopy overall, and reducing disparities in underserved communities. From a screening colonoscopy rate of only 42% in 2003, concerted efforts resulted in screening rates of 62% by 2007, and almost 70% in 2014, with the elimination of racial and ethnic disparities.^6^ This success was driven both by having city‐wide commitment and a patient navigation program that guides patients through pre‐colonoscopy preparation and provides culturally relevant support.

CRC remains a relatively underfunded disease in the US. The CDC provides five times as much funding annually per cancer death for breast and cervical cancer ($218 million) as for CRC ($43 million).^24^ Outside government, fewer resources are devoted annually to CRC control than to more publicized diseases; for example, Susan G. Komen provides $175 million toward breast cancer^25^ and the Amyotrophic Lateral Sclerosis (ALS) provides $38 million toward ALS,^26^ while the Alliance provides under $10 million toward CRC.^27^

This work was inspired by a desire to bridge these implementation and funding gaps. The novel concept was to develop a business‐mindset approach to provide the CRC advocacy community with a powerful fundraising tool, centered on a simple statement to prospective donors and philanthropists. We believe that the interventions we identified can be implemented broadly, and that the analyses presented and the business case developed in this work can be a powerful tool for donors who increasingly demand transparency into the return of a philanthropic investment.

We acknowledge some limitations. First, it is possible that not all unscreened persons will be equally amenable to being encouraged to screen. Thus, our activation cost estimates may apply to some initial fraction of the currently unscreened, but not to all. For instance, Kaiser Permanente Northern California has achieved impressive improvements in CRC screening rates, reaching 82% in the commercial population and 91% in the Medicare population, but neither are 100%.^5^ Second, as with any model, the assumptions can be challenged. We based our inputs on what we consider a balanced assessment of published literature.

We hope that our work will inspire regional partnerships to improve CRC screening rates, such as demonstration projects in geographies with large unscreened populations and low CRC screening rates, such as inner cities. Philanthropic efforts would be enhanced by donation of TV airtime for marketing campaigns, commitment by health systems and payors to help fund recurring activation costs for people using FIT, and donation of physician and facility services to provide free colonoscopies for unscreened people who are uninsured. At a national level, development of a navigation capability that could be accessed from anywhere would be a major achievement.

The focused message to potential philanthropists that “You can save a life from CRC with a $25,000 donation” could potentially reduce the US CRC deaths annually by tens of thousands.

## CONFLICT OF INTEREST

UL, advisor (UniversalDx, Lean), consultant (Covidien, Motus GI, Quorum, Clinical Genomics); others, none.

## AUTHOR CONTRIBUTION

Study concept and design, LD, AC, AK, UL; acquisition of data, AC, AK, UL; analysis and interpretation of data, AC, LD, AK, UL; drafting of the manuscript, AC, UL; critical revision of the manuscript for important intellectual content, AC, LD, AK, UL; statistical analysis, AC, UL; obtained funding, LD; technical, or material support, AC, LD, AK, UL; study supervision, LD, UL.

## Data Availability

Data sharing is not applicable to this article as no new data were created or analyzed in this study.
