# Supplementary material for: The case for philanthropic investment to increase colorectal cancer screening rates: A novel paradigm to address a public health challenge
Source: Cancer Med. 2019 Dec 5;9(3):1220–9. doi: 10.1002/cam4.2745 (PMC6997057; doi:10.1002/cam4.2745)
Supplement: Supplementary file 1 [file CAM4-9-1220-s001.docx]

**Appendix**

**The Case for Philanthropic Investment to Increase Colorectal Cancer Screening Rates:**

**A Novel Paradigm to Address a Public Health Challenge**

Ariel Carmeli,^1^ Lee Dranikoff,^2,3^ Arnab Kundu,^4^ Uri Ladabaum^5^

^1^Biden Cancer Initiative, Washington, D.C.; ^2^Colorectal Cancer Alliance Board of Directors, Washington D.C.; ^3^American Securities, New York, NY; ^4^Clarify Health Solutions, New York NY; ^5^Division of Gastroenterology and Hepatology, Stanford University School of Medicine, Stanford, CA

*Quantifying the potential public health impact of increases in Colorectal Cancer (CRC) screening*

The derivations of our estimates are detailed below, and illustrated in the flowchart in Appendix Figure 1.

SEER provided an estimate of ~13,900 annual CRC deaths among people aged less than 50 or greater than 84 years (Group A).

We estimated that ~6,900 annual CRC deaths result from interval cancers, of which all are not potentially addressable by improved screening; in addition, we estimated that ~4,400 CRC deaths result from screen-detected cancers, of which we estimated one-third or ~1,500 are not potentially addressable by improved screening. This sums to ~8,300 annual CRC deaths that are not potentially addressable by improved screening (Group B). This was derived as follows.

Interval cancers and deaths:

- There are ~65 million people aged 50-84 adherent with screening (given ~100 million people in this age range in the US),^1^ ~65% of whom are compliant with screening.^2^
- We assumed a rate of interval cancer of 5 cases per 10,000 person-years.^3^
- We estimate the deaths per interval cancer as 0.21, calculated from the 29 deaths out of 139 interval cancer cases in Quintile 3 from the same study.^3^
- Thus, 65 million * (5/10,000) * 0.21 = ~6,900 interval cancer deaths.

Screen-detected cancers:

- There are ~6.5 million people screened each year, estimated as 1/10 of the number of people compliant with CRC screening as calculated above.
- We estimated that ¼ of these people are first-time screeners (~1.6 million people), with the remaining ~4.9 million people being 2^nd^ or later screeners.
- We estimated a CRC rate of 65 per 9989 people screened with colonoscopy for first-time screeners.^4^
- We assumed a CRC of 1/3 compared with first screening rate in subsequent screens, given a standardized incidence ratio (SIR) of 0.2;^5^ this represented a conservative estimate.
- We estimated the death rate for screen-detected cancers as 21% based on multiplying mortality rates by stage (localized, regional, disseminated – 10%, 30%, 90%, respectively)^6^ and with the respective distribution of CRC at screening (localized, regional, disseminated – 62%, 31%, 6%, respectively).^4^
- Thus, in first-time screeners: 1.6 million people * (65 CRCs/9989 people screened) * 21% death rate per screen-detected CRC= ~2,200 annual deaths.
- Thus, in later screeners: 4.9 million people * (65 CRCs/9989 people screened) * (1/3 rate in subsequent screens) * 21% death rate per screen-detected CRC= ~2,200 annual deaths.
- The sum of screen-detected deaths in first time screeners and in later screeners is ~4,400 annual deaths. We estimated that some of these deaths are potentially addressable, those among people who were not compliant prior to their CRC screen, e.g., waited until age 55 for their first CRC screen. It has been studied that a 12-month delay in CRC screen beyond the guidelines results in a 3.2 odds ratio for advanced disease,^7^ and as such, we assume one-third of screen-detected deaths could not have been prevented or addressed by a CRC screening initiative and acknowledge this may be an optimistic assumption.

*Identifying barriers to screening and their relative contribution to screening non-adherence*

We identified the most significant barriers to adherence to CRC screening guidelines, and quantified the approximate magnitude for each barrier, based on a review of published literature and publicly available data from the American Cancer Society (ACS) and the Centers for Disease Control and Prevention (CDC) as shown in Appendix Table 1.

*Estimating the “activation cost” to overcome barriers and screen one additional person*

For each potential intervention, published literature and expert opinions were used to estimate the potential impact and cost (Appendix Table 2)

*Developing a holistic business case that is attractive to philanthropic organizations*

The potential mortality reduction with improved screening uptake was estimated as shown in Appendix Table 3. The absolute number of preventable deaths in a cohort of 1,000 persons were estimated as shown in Appendix Table 4.

Previously published literature supports our estimate of the benefit of screening (approximately an 84% reduction in mortality):

- A modeling analysis prepared for the Agency of Healthcare Research and Quality within the US Department of HHS suggests ~78-93% reduction in morality as a result of colonoscopy (1x per 10 years) or FIT (1x per year) from the ages of 50-85. This range reflects the outputs of three separate models: SimCRC, MISCAN, CRC-SPIN. Note that SimCRC and CRC-SPIN models alone would suggest a ~86-93% reduction in mortality.^8^
- A recent study of ~25,000 patients in the Veterans Affairs health system suggests that a colonoscopy reduces the risk of death from left-sided CRC by 72%.^9^
- The National Polyp Study from 1993 estimates ~76-90% reduction in CRC incidence as a result of colonoscopy use.^10^

Appendix Figure 1


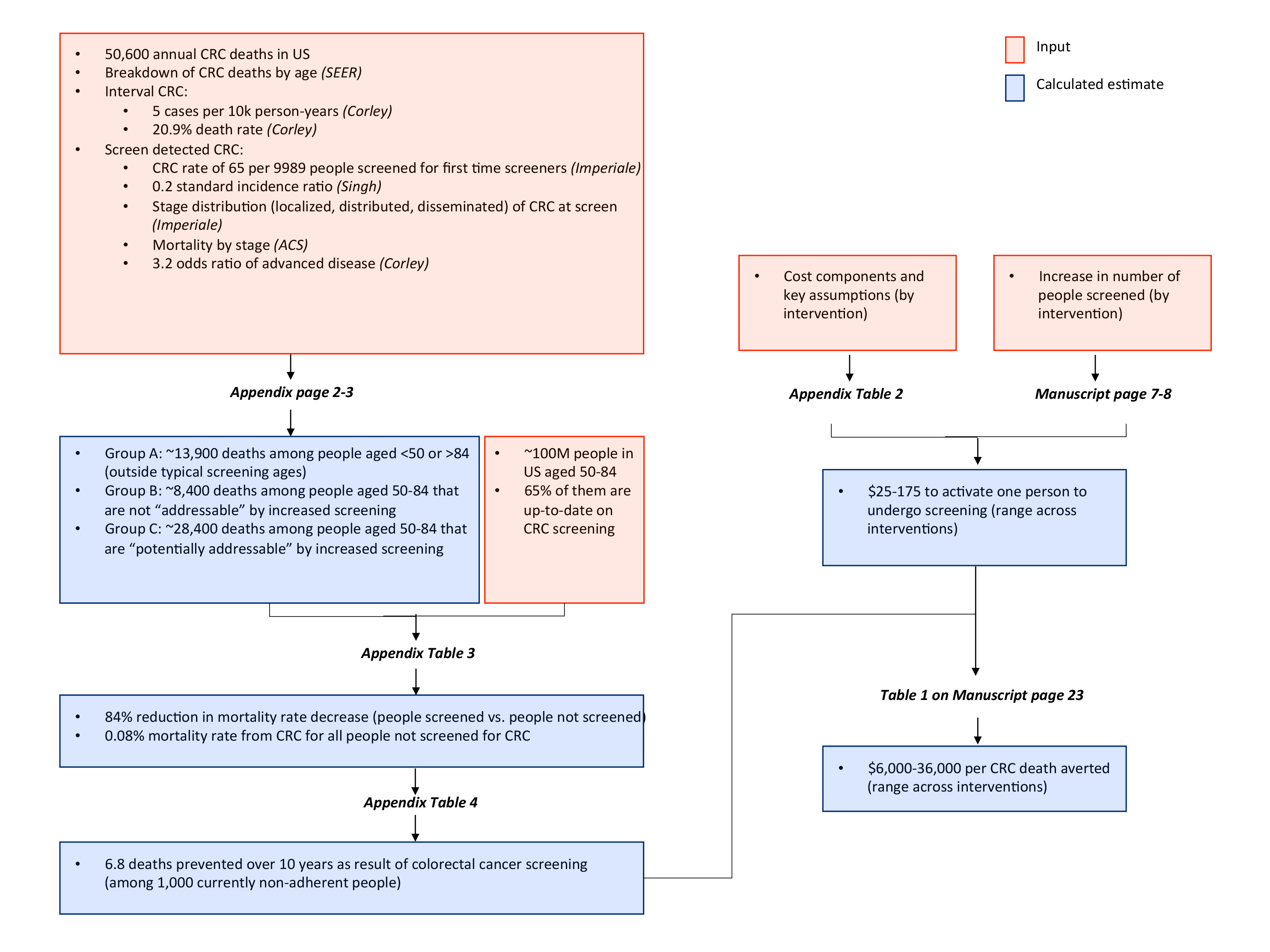


Appendix Table 1: Data sources used to estimate the magnitude of each barrier contributing to non-adherence to colorectal cancer screening guidelines in adults aged 50-74

| Phases of patient journey | Barrier | Estimate | Source |
| --- | --- | --- | --- |
| Pre-physician | Lack of insurance | ~25% of non-compliant patients are uninsured | National Colon Cancer Roundtable |
|  | Lack of patient awareness of CRC screening practices | ~10% of non-compliant patients are unaware of CRC screening | American Cancer Society |
|  | Logistical challenges | ~14-31% of non-compliant patients report logistic obstacles to getting screened (e.g., transportation problems, busy) | ^11^ |
| CRC screening recommendation | Lack of PCP recommendation for screening | ~21-48% of non-compliant patients report that they did not receive a CRC screening recommendation from their PCP | ^11, 12^ |
| Post recommendation through completion of CRC screening | Patient avoidance despite PCP recommendation | ~7-47% of non-compliant patients report avoidance as a barrier to screening (e.g., fear of cancer, not at risk due to lack of family history) | ^11^ |
|  | Fear/avoidance of colonoscopy preparation | ~7% of non-compliant patients report bowel prep as a barrier to colonoscopy | Centers for Disease Control and Prevention – Behavioral Risk Factor Surveillance System |
| Follow-up | Lack of timely subsequent screening after initial screening | ~20% of currently non-compliant patients were once screened for CRC | New York City Department of Health and Mental Hygiene |

CRC, colorectal cancer; PCP, primary care provider

Appendix Table 2: Summary of cost components and key assumptions for each intervention

| Intervention | Cost components | Key cost assumptions | Source |
| --- | --- | --- | --- |
| Marketing campaign | TV airtime | $30-50M | Screen for Life donated airtime; Centers for Disease Control and Prevention national Smoking Kills campaign |
| Patient navigation | Live navigation | $30 to navigate 1 patient | ^13^ |
|  | Program management/analytics | 1 person required per payor/ health system partner | Assumption |
|  | Call center set-up | 1 navigator required to navigate ~100 patients per week | New York City Department of Health and Mental Hygiene |
| FIT mailout (insured patients) | FIT mailing / reminders | $5 per person for mailing and robo-call reminders Cost of FIT kit is covered by insurance | Kaiser Permanente STOP CRC effort in Oregon |
|  | Follow-up patient navigation for colonoscopy | $30 to navigate 1 patient | ^13^ |
| Uninsured outreach | Patient acquisition | ~6,000 non-screened patients identified per safety net manager  ~$50,000 salary per safety net manager | Assumption |
|  | Cost to cover FIT kit | $5 per FIT kit | Kaiser Permanente |
|  | FIT mailing / reminders | $5 per person for mailing and robo-call reminders | Kaiser Permanente STOP CRC effort in Oregon |
|  | Cost to cover follow-up colonoscopy | ~$700-1,200 to cover 1 colonoscopy | Colon Cancer Alliance |
| High-touch health system engagement | Field force staff | ~9-12 PCPs per 1,000 unscreened patients  Each field force staff can visit 6 PCP per day  Each field force staff will visit each PCP 6 times per year  $125,000 salary per field force staff | Assumption |

FIT, fecal immunochemical test; PCP, primary care provider

Appendix Table 3. Estimated potential reduction in CRC mortality in currently non-adherent group

| **Population group** | **Population size** | **Annual CRC deaths** | **Mortality rate (deaths / population size)** | **Reduction in mortality rate vs. non-adherent group** |
| --- | --- | --- | --- | --- |
| Not addressable (Group B) | ~65 million | ~8,300 | ~0.013% | 84% |
| Potentially addressable (Group C) | ~35 million | ~28,400 | ~0.080% | N/A |

Appendix Table 4. Deaths prevented over 10 years as result of colorectal cancer screening (1,000 currently non-adherent people)

|  |  | **Calculation / Source** |
| --- | --- | --- |
| Number of potentially addressable deaths (per 10 years) | 8.0 | 10* annual number of potentially addressable deaths |
| Annual number of potentially addressable deaths | 0.80 | Number of people * Mortality rate |
| Number of people | 1,000 | Assumed |
| Mortality rate | 0.080% | From Appendix Table 3 |
|  |  |  |
| Reduction in mortality rate if addressed | 84% | From Appendix Table 3 |
|  |  |  |
| Deaths prevented (per 10 years) | 6.8 | Reduction in mortality rate (above) * Number of potentially addressable deaths (above) |

References

1. United States Census Bureau. Accessed at <https://www.census.gov> on September 25, 2018.

2. Sauer AG, Liu B, Siegel RL, Jemal A, Fedewa SA. Comparing cancer screening estimates: Behavioral Risk Factor Surveillance System and National Health Interview Survey. Prev Med. 2018;106: 94-100.

3. Corley DA, Jensen CD, Marks AR, et al. Adenoma detection rate and risk of colorectal cancer and death. N Engl J Med. 2014;370: 1298-1306.

4. Imperiale TF, Ransohoff DF, Itzkowitz SH, et al. Multitarget stool DNA testing for colorectal-cancer screening. N Engl J Med. 2014;370: 1287-1297.

5. Singh H, Turner D, Xue L, Targownik LE, Bernstein CN. Risk of developing colorectal cancer following a negative colonoscopy examination: evidence for a 10-year interval between colonoscopies. JAMA. 2006;295: 2366-2373.

6. American Cancer Society. Survival Rates for Colorectal Cancer. Accessed on February 10, 2019 at <https://www.cancer.org/cancer/colon-rectal-cancer/detection-diagnosis-staging/survival-rates.html>.

7. Corley DA, Jensen CD, Quinn VP, et al. Association Between Time to Colonoscopy After a Positive Fecal Test Result and Risk of Colorectal Cancer and Cancer Stage at Diagnosis. JAMA. 2017;317: 1631-1641.

8. Knudsen AB, Zauber AG, Rutter CM, et al. Estimation of Benefits, Burden, and Harms of Colorectal Cancer Screening Strategies: Modeling Study for the US Preventive Services Task Force. JAMA. 2016;315: 2595-2609.

9. Kahi CJ, Pohl H, Myers LJ, Mobarek D, Robertson DJ, Imperiale TF. Colonoscopy and Colorectal Cancer Mortality in the Veterans Affairs Health Care System: A Case-Control Study. Ann Intern Med. 2018;168: 481-488.

10. Winawer SJ, Zauber AG, Ho MN, et al. Prevention of colorectal cancer by colonoscopic polypectomy. The National Polyp Study Workgroup. N Engl J Med. 1993;329: 1977-1981.

11. Denberg TD, Melhado TV, Coombes JM, et al. Predictors of nonadherence to screening colonoscopy. J Gen Intern Med. 2005;20: 989-995.

12. DuBard CA, Schmid D, Yow A, Rogers AB, Lawrence WW. Recommendation for and receipt of cancer screenings among medicaid recipients 50 years and older. Arch Intern Med. 2008;168: 2014-2021.

13. Jandorf L, Stossel LM, Cooperman JL, et al. Cost analysis of a patient navigation system to increase screening colonoscopy adherence among urban minorities. Cancer. 2013;119: 612-620.
